# Selective Over-Expression of Endothelin-1 in Endothelial Cells Exacerbates Inner Retinal Edema and Neuronal Death in Ischemic Retina

**DOI:** 10.1371/journal.pone.0026184

**Published:** 2011-10-28

**Authors:** Simon S. F. Cheung, Justin W. C. Leung, Amy K. M. Lam, Karen S. L. Lam, Stephen S. M. Chung, Amy C. Y. Lo, Sookja K. Chung

**Affiliations:** 1 Department of Anatomy, The University of Hong Kong, Hong Kong, China; 2 Department of Medicine, The University of Hong Kong, Hong Kong, China; 3 Department of Physiology, The University of Hong Kong, Hong Kong, China; 4 Research Centre of Heart, Brain, Hormone and Healthy Aging, Li Ka Shing Faculty of Medicine, The University of Hong Kong, Hong Kong, China; Chinese University of Hong Kong, Hong Kong

## Abstract

The level of endothelin-1 (ET-1), a potent vasoconstrictor, was associated with retinopathy under ischemia. The effects of endothelial endothelin-1 (ET-1) over-expression in a transgenic mouse model using Tie-1 promoter (TET-1 mice) on pathophysiological changes of retinal ischemia were investigated by intraluminal insertion of a microfilament up to middle cerebral artery (MCA) to transiently block the ophthalmic artery. Two-hour occlusion and twenty-two-hour reperfusion were performed in homozygous (Hm) TET-1 mice and their non-transgenic (NTg) littermates. Presence of pyknotic nuclei in ganglion cell layer (GCL) was investigated in paraffin sections of ipsilateral (ischemic) and contralateral (non-ischemic) retinae, followed by measurement of the thickness of inner retinal layer. Moreover, immunocytochemistry of glial fibrillary acidic protein (GFAP), glutamine synthetase (GS) and aquaporin-4 (AQP4) peptides on retinal sections were performed to study glial cell reactivity, glutamate metabolism and water accumulation, respectively after retinal ischemia. Similar morphology was observed in the contralateral retinae of NTg and Hm TET-1 mice, whereas ipsilateral retina of NTg mice showed slight structural and cellular changes compared with the corresponding contralateral retina. Ipsilateral retinae of Hm TET-1 mice showed more significant changes when compared with ipsilateral retina of NTg mice, including more prominent cell death in GCL characterized by the presence of pyknotic nuclei, elevated GS immunoreactivity in Müller cell bodies and processes, increased AQP-4 immunoreactivity in Müller cell processes, and increased inner retinal thickness. Thus, over-expression of endothelial ET-1 in TET-1 mice may contribute to increased glutamate-induced neurotoxicity on neuronal cells and water accumulation in inner retina leading to edema.

## Introduction

Diabetic retinopathy (DR) and other ocular diseases in diabetes, such as central retinal artery occlusion (CRAO) and glaucoma, is thought to be the consequence of retinal ischemia, leading to visual impairment and blindness [1]. Retina undergoing ischemia displayed a number of pathological and cellular changes. Reactivity of glial cells are activated by up-regulated expression of glial fibrillary acidic protein (GFAP) [2], with the release of massive amounts of glutamate from injured neurons which is suggested to be neurotoxic [3]. Neuronal cell death was observed by the presence of pyknotic nuclei, especially in the cells in ganglion cell layer (GCL) [4], [5], [6], [7], [8]. Moreover, increased extracellular water transport and accumulation was present in inner retina, which is characterized by increased extracellular fluid volume, increased aquaporin-4 (AQP-4) immunoreactivity and swelling of retinal glial cells, leading to inner retinal edema [9].

Endothelin-1 (ET-1), a 21-amino acid secretory protein synthesized in vascular endothelial cells, is a potent vasoconstrictor. A possible link between elevated plasma ET-1 level and retinopathy under ischemia has been established. Administration of ET-1 into the posterior vitreous body or the optic nerve of animal models led to physiological and cellular damages of ischemic insult, including obstruction of retinal blood flow, elevated scotopic b-wave in electroretinogram and apoptosis of cells in GCL [10], [11], [12]. This is because the increased ET-1 concentration would elevate vitreous glutamate level [13] and augmented activities and responses to glutamate in the nuclei of the solitary tract (NTS) neurons [14], which may increase the excitotoxic effects of glutamate to neuronal cells. The hypertensive property of ET-1 was suggested to play a role in ischemic insult in retina.

In order to study the pathogenic changes of ET-1 to ischemic stress in central nervous system, a hypertensive transgenic mouse model with over-expression of ET-1 in vascular endothelial cells using Tie-1 promoter (TET-1 mice) has been generated [15]. It has been shown that a more severe neurological deficit, larger brain infarct size and infarct volume following transient middle cerebral artery occlusion (MCAO) was present in homozygous (Hm) TET-1 mice, indicating that over-expressing ET-1 in endothelial cells is deleterious on neuronal survival after ischemic conditions [16]. This transgenic mouse model has the advantage over other ischemic models of ET-1 by regulating expression of ET-1 level endogenously in a specific cell type (endothelial cells) rather than regulating its level by external administration to induce the respective effects under ischemia. The present study is to further investigate the effects of endothelial ET-1 in retinopathy after transient inner retinal ischemia and reperfusion of ophthalmic artery (OA) and central retinal artery (CRA) [17]. We hypothesize that TET-1 mice may induce more severe ischemia-related cellular damages and edema in retina after retinal ischemia induced by OA occlusion.

## Materials and Methods

### Ethics Statement

The use of animals in this study was conducted according to the requirements of the Cap. 340 Animals (Control of Experiments) Ordinance and Regulations, and all relevant legislation and Codes of Practice in Hong Kong. All the experimental and animal handling procedures were approved by the Faculty Committee on the Use of Live Animals in Teaching and Research in The University of Hong Kong (Permit number #634-01).

### Generation of TET-1 transgenic mice

Male heterozygous (He) TET-1 mice were generated by microinjection of the ET-1 construct, which contains the mouse ET-1 cDNA with SV40 polyA driven by the Tie-1 promoter [16], [18]. TET-1 mice were maintained in the F1 hybrid background (C57BL/6J×CBA). Animals were kept under controlled environmental conditions with respect to temperature (19°C), humidity (55%), and 12-hour light-dark schedule. They received sterilized water and mouse diet *ad libitum*.

### Semi-quantitative reverse transcriptase-PCR (rt-PCR) of ET-1 gene

Mice at the age of 6 to 8 weeks were sacrificed, their eyes were cut underneath the optic nerve and the retinae were dissected under dissecting microscope (Stemi SV6; Carl Zeiss, Thornwood, NY), frozen in liquid nitrogen and stored in a −80°C. Retinal tissues were homogenized in 1 ml ice-cold TRI REAGENT® (Molecular Research Center, Cleveland, Ohio) and the total RNA from retina was isolated according to manufacturer's protocol. 1.5 µg of total RNA was treated with DNase I (Boehringer, Ingelheim, Germany) and reverse transcription was performed with Oligo(dT)18, Superscript™ II RNase H- reverse transcriptase (Invitrogen, Carlsbad, CA), DTT and dNTP mixture. The primer sequences and condition for rt-PCR of ET-1 has been previously mentioned [19]. The mouse GAPDH gene was co-amplified in the same reaction as internal control. The ratio of intensities of ET-1 to GAPDH genes expression of each sample was determined by densitometer.

### Transient retinal ischemia induced by occlusion of ophthalmic artery

Age-matched Hm TET-1 and non-transgenic (NTg) mice at the age of 8 to 10 weeks were subjected to retinal ischemia as previously described [20], [21], [22], [23]. In brief, animals were first anesthetized (2% halothane in 70% N_2_O/30% O_2_ for induction and 1% halothane in 70% N_2_O/30% O_2_ for maintenance) and the rectal temperature was kept at 37°C. An intraluminal insertion of a nylon microfilament was inserted into the right external carotid artery through internal carotid artery (ICA) up to the middle cerebral artery (MCA). This method could block the cerebral blood flow from the right ICA to OA and CRA, leading to ischemia of the right retina (ipsilateral side). The drop in retinal blood flow was indirectly monitored by laser Doppler flowmetry (Perimed, Järfälla, Sweden) where an optic fibre was placed on the skull to measure the relative regional blood flow in the core territory of the right middle cerebral artery in the brain. Anaesthesia was maintained for another 5 min after ischemia induction to ensure that the filament maintained its position inside the vessel. The wound was then closed and the animal was released from anaesthesia. Fifteen minutes before reperfusion, animals were anesthetized again as above during which the filament was removed at 2 hrs after ischemia induction. Reperfusion was then allowed for 22 hours. The mice were then sacrificed to enucleate the right and left eyeballs, which represented the ipsilateral (ischemic) and the contralateral (non-ischemic) side, respectively. A suture (tied onto the upper conjunctiva at the time of collection) was used for identification of orientation. This suture was then used during paraffin embedding to identify the superior retina. They were fixed in 4% paraformaldehyde overnight at 4°C, dehydrated with a graded series of ethanol, xylene and then embedded in paraffin wax. With the placement of the eyeball horizontally, the nasal and temporal parts were identified on either side of the paraffin block.

### Histological and immunocytochemical techniques

Serial paraffin sections of 7 µm thick from non-ischemic and ischemic eyeballs were prepared using the microtome (Microm HM315, Germany). The sections were deparaffinized in xylene, rehydrated with a graded series of ethanol, and stained briefly with hematoxylin and eosin (Sigma-Aldrich, St. Louis, MO). To perform immunocytochemistry (ICC) of GFAP, glutamine synthetase (GS) and AQP-4, sections of ischemic and non-ischemic retinae were blocked with 1.5% normal goat serum (Vector Laboratories, San Francisco, CA) for 1 hour, followed by incubation in diluted primary antibodies overnight at 4°C. Incubation of blocking serum served as control. The primary antibodies and their concentrations were rabbit anti-GFAP (1∶2000; Dako, Carpinteria, CA), rabbit anti-AQP-4 (1∶ 800; Chemicon International, Temecula, CA) and rabbit anti-GS (1∶ 300; Santa Cruz Biotechnology, Santa Cruz, CA). The sections were subsequently incubated with biotinylated goat anti-rabbit secondary antibody (Vector Laboratories), and immunoreactive signals were visualized by incubation with the avidin-biotin-peroxidase complex (Vector Laboratories) for 30 min and diaminobenzidine tetrahydrochloride (Zymed Laboratory, San Francisco, CA) for 2 min. Finally, the sections were counterstained with hematoxylin, dehydrated, cover-slipped and mounted with mounting medium.

### Quantitation of cell death in retinal ganglion cell layer

Retinal sections containing the optic nerve that were stained with hematoxylin and eosin were chosen for viewing. Two images, one on either the nasal or temporal side, were taken at a region about 300 µm away from the optic nerve using the 20× objective (Olympus IX71, Olympus, Japan) as previously described [21], [22], [23]. Cells with pyknotic nuclei and the total number of cells in GCL in the images were counted. It was assumed that the percentage of pyknotic nuclei represented the percentage of cell loss in the GCL. The severity of cell death in GCL was classified into four levels: “severe” represented more than 80% of cells in GCL on the section had pyknotic nuclei, “moderate” represented 20% to 80% of these cells had pyknotic nuclei, less than 20% of cell death was classified as “mild”, and absence of pyknotic nuclei was defined as “none”. Sections from 12–14 animals in each group (n = 12–14) were analyzed.

### Measurement of inner retinal thickness

Digital photographs were taken using 20× objectives from the nasal and temporal sides (about 300 µm from the optic nerve) in each of the contralateral and ipsilateral retinae. In each photo, the thickness of three regions in the retina was measured: inner nuclear layer (INL), from inner plexiform layer (IPL) to inner limiting membrane (ILM), and from INL to ILM representing the thickness of the whole retina [24]. Data from each mouse was taken by averaging two measurements from nasal and temporal sides of retina, respectively.

### Statistical analysis

All data were expressed as means ± SEM. Statistical analyses were performed using one-way ANOVA with Bonferroni's post hoc tests, Fisher's exact test, Mann-Whitney Test, and Kruskal-Wallis Test by Statistical Package for the Social Sciences software (SPSS 12.0, SPSS). Difference with *p* value<0.05 was considered statistically significant.

## Results

### Up-regulated mRNA and peptide expressions of ET-1 in TET-1 mice

**Figure 1:**
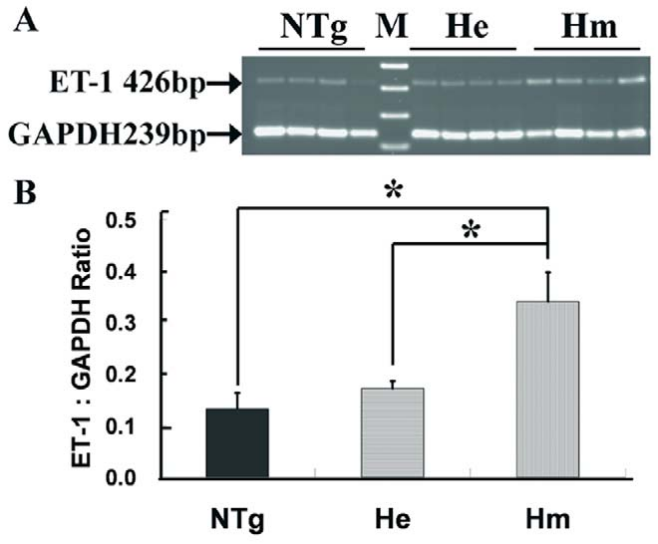
Semi-quantitative rt-PCR analysis showing total ET-1 mRNA expression in the retinae of non-transgenic (NTg), heterozygous (He) and homozygous (Hm) TET-1 mice. A: Ethidium bromide-stained agarose gels of rt-PCR products of ET-1, showing increased ET-1 mRNA expression in transgenic mouse retina. The molecular size marker (M) was 1 kb plus marker. B: Histograms showing the levels of ET-1 mRNA expression normalized to that of GAPDH. n = 4 for each experimental group. *: *p*<0.05 (Kruskal-Wallis Test).

**Figure 2:**
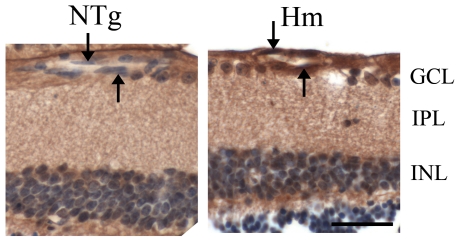
Immunohistochemical staining of ET-1 antibody. Immunoreactivity of ET-1 was up-regulated in the endothelial cells (arrows) of Hm TET-1 mice compared with the corresponding NTg mice. Scale bar = 25 µm.

Total ET-1 mRNA expression in 6- to 8-week-old in Hm TET-1 mouse retina was about 2.5 times higher than that of NTg mice by semi-quantitative rt-PCR (Fig. 1), which was statistically significant (*p*<0.05, Kruskal-Wallis Test). This showed that over-expression of ET-1 could be observed in retina at the transcription level. Peptide expression of ET-1 was also studied in the retinal sections of 6-week-old TET-1 mouse retina by ICC using rabbit anti-ET-1 antibody. Immunopositive signal of ET-1 was localized in the nerve fibres of ILM, endothelial cells and cells in INL (Fig. 2). ET-1 peptide expression was much higher in the endothelial cells of Hm TET-1 mice when compared with that of NTg mice.

### More severe cell death in retinal ganglion cell layer in TET-1 mice

Cellular morphology in retinal sections of ischemic and non-ischemic retinae of NTg and Hm TET-1 mice were examined. Similar to our previous results, there was presence of cells with pyknotic nuclei in the GCL [21], [22], [23]. We then strictly followed the previously published protocol in counting these cells with pyknotic nuclei and the total number of cells in the GCL. The severity of cell death in GCL was classified according to the percentage of pyknotic nuclei, which represented the percentage of cell loss in the GCL. Our quantitation showed that all contralateral retinae in NTg and Hm TET-1 mice were classified as “none”. “Severe” cell death in GCL was seen in more than half (8 out of 14) of the ipsilateral retinae of Hm TET-1 mice, while the others were classified as either “moderate” (2 out of 14) or “mild” (4 out of 14). On the other hand, only 1 out of the 12 samples in the ipsilateral retina of NTg mice had “severe” cell death in GCL, while “mild” death was observed in 7 out of the 12 mice (Table 1). A significant difference in the severity of cell death in GCL between ipsilateral retinae of NTg and Hm TET-1 mice was observed by comparing the number of retinae with “severe” changes and with “moderate or mild” changes (*p*<0.05, Fisher's exact test).

**Table 1:** Severity of the presence of pyknotic nuclei in cells in ganglion cell layer (GCL) after transient inner retinal ischemia.

	*None*	*Mild*	*Moderate*	*Severe*	*Non-severe (Moderate+Mild)*	*Total*
***NTg Contralateral***	12	0	0	0	12	12
***NTg Ipsilateral***	0	7	4	1	11	12
***Hm Contralateral***	14	0	0	0	14	14
Hm Ipsilateral*	0	4	2	8	6	14

### Increased GFAP immunoreactivity in ischemic retina

**Figure 3:**
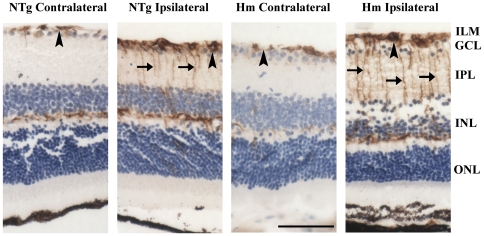
Immunohistochemical staining of glial fibrillary acidic protein (GFAP). Compared with the contralateral retinae of NTg and Hm TET-1 mice, GFAP signal was up-regulated in the ipsilateral retinae in the astrocytes around capillaries in the inner limiting membrane (arrowheads) and the Müller cell processes in IPL (arrows). n = 5 for each experimental group. Scale bar = 50 µm.

Glial cell reactivity in the ipsilateral and contralateral retinae of NTg and Hm TET-1 mice was studied by immunocytochemical staining of GFAP (Fig. 3). In the contralateral retinae of both genotypes, GFAP was mainly expressed in the astrocytes and Müller cell endfeet in outer plexiform layer (OPL), with a few radial processes of Müller cells running proximally in IPL. The intensity of signal in Hm TET-1 and NTg mice in the contralateral retinae was similar. In the ipsilateral retinae of both genotypes, the intensity of GFAP immunoreactivity was dramatically increased in the Müller cell processes and astrocytes around the retinal capillaries when compared with their corresponding contralateral retinae. The expression was relatively stronger in the peripheral and central regions. Yet, a slightly stronger intensity was observed in the ipsilateral retina of Hm TET-1 mice than that of NTg mice. This concurred with the result of more neuronal cell death observed in the ipsilateral retina of Hm TET-1 mice.

### Increased glutamine synthetase immunoreactivity in the Müller cells of TET-1 ischemic retina

**Figure 4:**
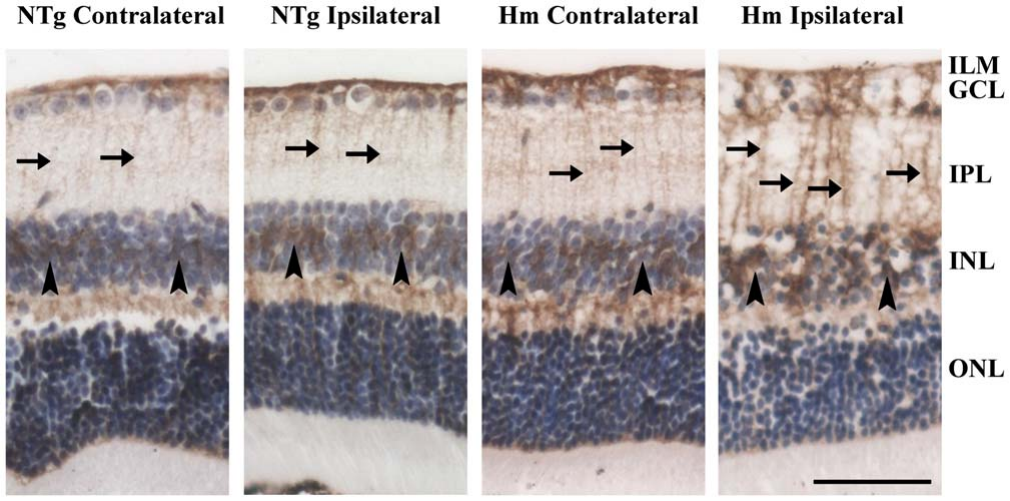
Immunohistochemical staining of glutamine synthetase (GS) antibody. Up-regulation of GS signal was found in the Müller cell processes in IPL (arrows) and Müller cell bodies in INL (arrowheads) in the ipsilateral retina of Hm TET-1 mice compared with the contralateral retina of Hm TET-1 mice and the ipsilateral retina of NTg mice. n = 5 for each experimental group. Scale bar = 50 µm.

In the non-ischemic retinae of NTg and Hm TET-1 mice, immunoreactivity of GS was mainly present in the astrocytes, Müller cell bodies in INL and Müller cell processes from ILM to OLM (Fig. 4). The expression of GS in the peripheral and central retinal regions was similar. Slightly increased immunoreactivity was observed in the Müller cell processes in the ipsilateral retina of NTg mice when compared with the corresponding contralateral retina, as well as in the contralateral retina of Hm TET-1 mice when compared with the contralateral retina of NTg mice. A significant up-regulation of GS expression was also observed in the Müller cell processes and Müller cell bodies in the ipsilateral retina of Hm TET-1 mice when compared with the contralateral and ipsilateral retinae of NTg mice.

### Increased aquaporin-4 immunoreactivity in the glial cells of TET-1 mice

**Figure 5:**
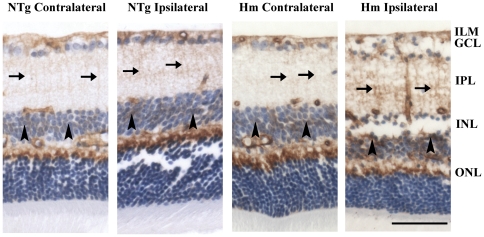
Immunohistochemical staining of aquaporin-4 (AQP4). Up-regulation of AQP4 signal was found in the Müller cell processes in IPL (arrows) and Müller cell bodies in INL (arrowheads) in the ipsilateral retina of Hm TET-1 mice compared with the corresponding contralateral retina, while similar intensity of AQP4 signal was present in the contralateral and ipsilateral retinae of NTg mice. n = 5 for each experimental group. Scale bar = 50 µm.

Expression of a water transport protein, aquaporin-4 (AQP4), was determined for the presence of edema and water accumulation of glial cells in ischemic retina. In the contralateral retinae of both NTg and Hm TET-1 mice, positive immunoreactivity of AQP4 was found in the Müller cell processes from ILM to OLM, astrocytes around blood vessels in GCL, Müller cell bodies in INL and astrocytic endfeet in OPL (Fig. 5). Similar findings were observed in a recent immunohistochemical study [25]. The expression of AQP4 in the peripheral and central regions was similar. Contralateral and ipsilateral retinae of NTg mice presented a similar pattern and intensity of AQP4 immunoreactivity, as well as between the contralateral retinae of NTg and Hm TET-1 mice. However, a significant up-regulation of immunoreactive signal was observed in the Müller cell processes and Müller cell bodies of the ipsilateral retina of Hm TET-1 mice compared with the corresponding contralateral retina of Hm TET-1 mice.

### Increased inner retinal thickness in TET-1 mice after transient ischemia

According to our semi-quantitative observation, the major phenotype of ischemic injury in the ipsilateral retina of Hm TET-1 mice was “severe” while that of NTg mice was “mild”. Therefore, representative photomicrographs from each experimental animal group were selected after careful screening for retinal thickness measurement. Similar thicknesses of INL, IPL to ILM and inner retinal layer were found between the contralateral retinae of Hm TET-1 and NTg mice (Table 2), as well as between the ipsilateral and contralateral retinae of NTg mice. On the contrary, there was a significant increase in the thickness of IPL to ILM and inner retina in the ipsilateral retina of Hm TET-1 mice when compared with the contralateral retina of Hm TET-1 mice (*p*<0.05, Mann-Whitney Test), while similar INL thickness was found between the two groups. Therefore, the increase in inner retinal thickness was solely due to the increase in the thickness from IPL to ILM.

**Table 2:** Thicknesses of inner nuclear layer (INL), inner plexiform layer (IPL) to inner limiting membrane (ILM) and the whole inner retina (µm).

	*Thickness IPL+ILM*	*Thickness of INL*	*Thickness of inner retina*
***NTg Contralateral***	57.2±3.5	29.8±0.8	87.0±2.8
***NTg Ipsilateral***	61.5±3.5	30.5±1.9	92.0±5.2
***Hm Contralateral***	56.2±3.1	28.9±1.8	85.2±3.9
***Hm Ipsilateral***	71.7±3.9*	29.0±1.9	100.6±3.3*

## Discussion

Mild inner retinal ischemia was induced by MCAO in mice with an absence [20] or a small number of apoptotic neurons [17]. As indicated by Kaja et al, this model is able to recapitulate the cellular and molecular changes in the retina after stroke. This is particularly clinically relevant since acute thrombotic/embolic stroke and transient ischemic attack in humans are often associated with temporary diminishment (ie, amaurosis fugax) or even permanent loss of vision [26].

In this study, the ischemic model induced by middle cerebral artery occlusion also resulted in a mild transient retinal ischemia with neuronal cell damage in mice with F1 hybrid background, as suggested by increased GFAP immunoreactivity in the Müller cell processes and astrocytes, and the presence of pyknotic nuclei in the cells in GCL in the NTg ipsilateral retinae. Thus, slight cellular and structural changes were present in the NTg ipsilateral retinae, including pyknotic nuclei were present in less than 80% of cells in GCL, slightly increased GS expression in the Müller cell processes and no significant changes in AQP4 immunoreactivity and inner retinal thickness. On the contrary, more severe cellular and structural changes were observed in the ipsilateral retina of Hm TET-1 mice, including presence of pyknotic nuclei in more than 80% of cells in GCL, prominent up-regulated GS expression in Müller cell processes, up-regulation of AQP-4 immunoreactivity and significant increase in inner retinal thickness. Under these prominent cellular changes observed in the ischemic retina, we may deduce that Hm TET-1 mice with over-expression of ET-1 in endothelial cells would exacerbate the effects of neuronal cell death and retinal edema after OA occlusion. Previous studies already showed significant loss of cells in GCL layer following intraocular injection of ET-1 to the optic nerve [11], [27], increased brain swelling and water contents in animals injected with ET-1 [28] and in transgenic mice with over-expressing ET-1 in astrocytes using GFAP promoter after transient MCAO [29], suggesting a possible correlation between induction of ET-1 to neuronal cell death and edema.

Down-regulation of GS expression has been shown in Müller cells after ischemia and reperfusion [13]. An increase in GS expression in glial cells was thus suggested to be neuroprotective with decreased glutamate neurotoxicity. However, our experimental results showed exacerbated immunoreactivity of GS in the Müller cell processes of Hm TET-1 mice, followed by more severe neuronal cell death. We propose an alternate mechanism for more severe neuronal cell death. Primary culture study on fetal murine cortical neurons showed that GS has relatively low affinity to neuronal and glial glutamate transporters, suggesting that GS may not be capable of lowering glutamate levels below the threshold of glutamate toxicity [30]. Previous studies suggested that increased ET-1 expression would up-regulate glutamate level in the vitreous after optic nerve ischemia [13], as well as the activities and responses to glutamate in NTS neurons [14], increased mRNA expression of endogenous ET-1 in TET-1 mice may enhance extracellular glutamate level in retina. During ischemic stress, the glutamate level was so high that the low-affinity GS could not reduce the glutamate level below the threshold of glutamate neurotoxicity. ET-1 itself would then impose an augmented response to glutamate-induced neurotoxicity, more pyknotic nuclei were present in the cells of GCL in Hm TET-1 ischemic retina. Further investigation is required to study the activities and roles of extracellular glutamate, GS and ET-1 to glutamate neurotoxicity in retina through the TET-1 mouse model.

AQP4 is the main water channel protein which regulates the bi-directional movement of water across membranes and involved in the maintenance of the ionic and osmotic balance [31]. Increased AQP4 expression during ischemia would promote neuronal cell swelling with over-excitation of glutamate receptors, leading to neuronal cell depolarization. Glial cell swelling occurs during reperfusion, with down-regulation of functional K^+^ channels and reduced transmembrane inwardly rectifying K^+^ (Kir) currents [32]. Therefore, ischemia-reperfusion process would increase extracellular fluid volume and swelling of retinal glial cells, contributing to progressive thickening of inner retina and development of macular edema [9], which consequently contributes to photoreceptor degeneration and neuronal cell death [33]. Possible involvement of AQP4 in ET-1-induced edema has been suggested by increased brain swelling and water contents in transgenic TET-1 mice [16] and in another transgenic mouse model with over-expression of ET-1 in astrocytes using GFAP promoter [29] following transient MCAO. The results further proved that over-expression of endothelial ET-1 in Hm TET-1 mice may promote water accumulation and edema in the glial cells of inner retina, leading to more severe neuronal cell death after ischemia and reperfusion.

This study showed that the transgenic mice model with over-expression of endothelial ET-1 may enhance the effects of glutamate-induced neurotoxicity after induction of inner retinal ischemia and reperfusion with exacerbated neuronal cell death. Moreover, TET-1 mice may also contribute to increased water accumulation in the glial cells, leading to retinal edema with increased inner retinal thickness. The TET-1 mouse model after mild transient inner retinal ischemia may serve as a suitable model to study pathological changes in ischemic retinopathy simulating disease conditions like DR, glaucoma, peripheral or central ischemia which may lead to ocular ischemia or stroke, and is useful for therapeutic studies on these disorders.
